# Adsorption of methylene blue on an agro-waste oiltea shell with and without fungal treatment

**DOI:** 10.1038/srep38450

**Published:** 2016-12-05

**Authors:** Jiayang Liu, Enzhong Li, Xiaojuan You, Changwei Hu, Qingguo Huang

**Affiliations:** 1 Fermentation Technology Division, School of Bioengineering, Huanghuai University, Zhumadian 463000, China; 2 Shandong Provincial Key Laboratory of Water and Soil Conservation and Environmental Protection, Linyi University, Linyi, 276000, China; 3 Department of Crop and Soil Sciences, University of Georgia, Griffin, Georgia 30223, United States

## Abstract

A lignocellulosic waste oiltea shell (OTS) was evaluated as an inexpensive sorbent to remove methylene blue (MB) from aqueous solution. Fungal treatment of OTS increased the MB adsorption by modifying the physicochemical properties of OTS and simultaneously produced laccase as a beneficial co-product. Without fungal treatment, the maximum amount of adsorption (*q*_m_) of MB by OTS was 64.4 mg/g, whereas the treatment with fungus *Pycnoporus* sp. and *Trametes versicolor* increased *q*_m_ up to 72.5 mg/g and 85.7 mg/g, respectively. This is because of the improved surface area and pore sizes as well as altered chemical compositions. The equilibrium sorption data for OTS both with and without treatment fitted to the Langmuir model, and the sorption rate data well fitted to the pseudo second-order kinetic model. The changes in free energy (Δ*G*°) and separation factor (*R*_*L*_) indicated that the sorption was spontaneous and favorable. Scanning electron microscopy and Fourier transform infrared spectroscopy showed the changes in the surface morphology and functional groups of OTS after fungal treatment. The agro-waste OTS could be utilized as a low-cost adsorbent for efficient dye removal, and fungal treatment can serve as a mild and clean technique to increase the adsorptive capacity of OTS.

Highly efficient removal of synthetic dyes from wastewater has been attempted by various methods. Among them, adsorption is a promising one because of its low cost and high efficiency, particularly for biomass sorbents derived from agricultural residues^1^. Some lignocellulosic materials with rough surfaces, pores of various sizes, and active functional groups on their surface may have a great potential as adsorbents, and they are abundant in nature^2^. The adsorptive capability of lignocellulosic materials to environmental protection via adsorbing diverse organic and inorganic pollutants from aqueous solution to clean wastewater has been widely studied^2^. To date, diverse plant materials or their modified forms have been explored as biosorbents for removal of metal ions or organic pollutants from aqueous solution, such as corn stover, almond shell, banana peel, and *Saccharum bengalense*^1^,^3^,^4^.

Oiltea camellia extracts (OCE) have become a popular functional food, because of their significant effect on preventing obesity by inhibiting fatty acid synthase activity and adipogenesis^5^. Moreover, tea oil derived from oiltea seed has been used as cooking oil over thousands of years in China; it contains abundant unsaturated fatty acids^5^. Oiltea shell (OTS) accounts for ∼60% of the camellia fruit on a wet weight basis^5^. In southern China where oiltea camellia is widely planted, large amounts of OTS are produced, most of them are discarded as wastes; only a very limited fraction is reutilized as a culture medium for the mushroom industry. As a lignocellulosic material, OTS has a great potential to be used as a low-cost adsorbent^6^, but yet to be systematically evaluated.

Some treatment approaches, mainly physical and chemical, have been used to enhance the sorption capacity of raw lignocellulosic materials^7^,^8^,^9^. Biological treatment of biomaterials such as fungal cultivation is one promising method that has been assessed for other purposes including enhanced saccharification and modified animal feed^10^,^11^,^12^, but it has not been used for biosorbent modification. White-rot fungi are well known in natural carbon cycling via degrading lignocellulose using their powerful ligninolytic system and with the help of mycelial penetration into plant cell walls^13^. During fungal pretreatment of plant materials, beneficial enzymes are usually simultaneously secreted as co-product^10^,^12^,^14^, and these enzymatic extracts have been recently found to be useful in some promising fields, such as alleviation of soil water repellency (SWR)^15^, reducing excessive organic thatch layers on turfgrass greens^16^, decolorization of dyes^17^, and enhancement of lignocellulose saccharification for bioethanol production^18^.

This work aims to evaluate the potential of OTS as a novel biosorbent for dye removal, and the adsorption capacity of the OTS before and after treatment with a fungal culture using methylene blue (MB) as the model sorbate. To the best of our knowledge, this is the first report on the use of OTS as a biosorbent for dye removal, as well as the use of fungal cultivation as a treatment approach to enhance the adsorption capacity of a biosorbent.

## Methods

### Fungi, OTS, and MB

**Figure 1:**
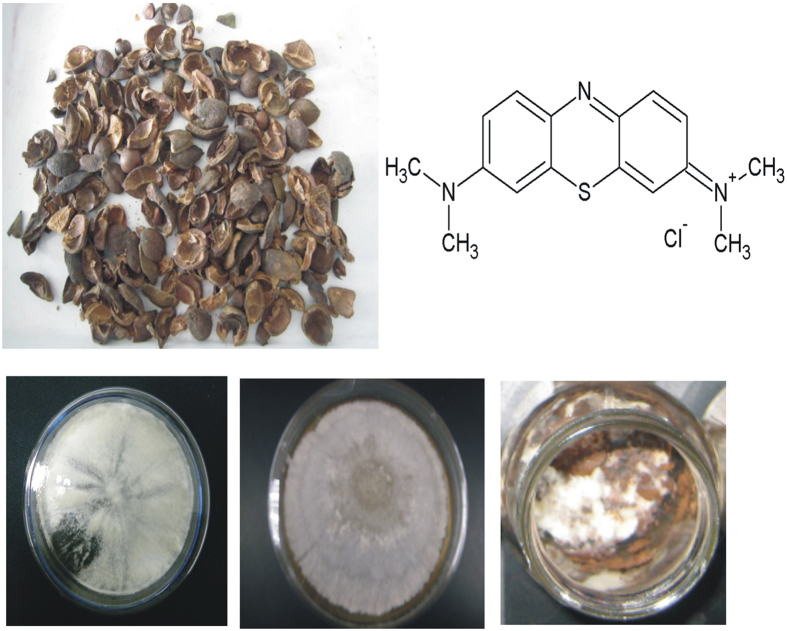
Experimental materials in this study. (upper left: air-dried OTS; upper right: chemical structure of MB; lower left: *Trametes versicolor* SYBC-L19; lower middle: *Pycnoporus* sp. SYBC-L3; lower right: fungal cultivation on OTS.)

Two typical white-rot fungi, namely *Pycnoporus* sp. SYBC-L3 (GenBank access number JX861099) and *Trametes versicolor* SYBC-L19 (GenBank access number JX861099), were employed in this study for the treatment of oiltea shell (OTS) (Fig. 1). *Pycnoporus* sp. SYBC-L3 is a robust laccase producer in a nutrition-limited medium^19^ or an optimized medium at bench or reactor scale^20^. *T. versicolor* SYBC-L19 is a newly isolated fungus with great potential in laccase production using water hyacinth as culture substrate^21^. The two fungi were maintained on potato dextrose agar (PDA) Petri dish at 4 °C and activated at intervals of two weeks.

OTS was collected from a planting base of oiltea camellia in Jiangxi Province in southern central China and washed with distilled water to remove the dirt and then air-dried (Fig. 1). The washed OTS was ground to pass a 10-mesh sieve and oven-dried at 40 °C to constant weight and then stored at room temperature prior to use. A basic dye methylene blue (MB9140, Sigma-Aldrich) was used as a model adsorbate to study adsorption behavior of OTS with or without fungal treatment. MB was dissolved in deionized water to varied concentrations as required in each batch experiment. The standard curve of OD_663 nm_ versus MB concentration was plotted to determine the dye content in the subsequent experiments^22^. Other chemicals used in this study were of analytical grade and locally purchased.

### Culture conditions

The solid-state culture medium containing OTS and 10% tap water was autoclaved at 121 °C for 20 min prior to fungal inoculation. Fungal cultivation was carried out in wide-mouth bottles with a height of 10 cm and a diameter of 5 cm, thus allowing good oxygen circulation (Fig. 1). Each bottle with 20 g OTS was inoculated with five PDA disks (diameter of 5 mm) cut from the margins of actively growing fungal colony. To avoid microbial contamination, an eight-layer gauze was used to cap each bottle and fastened with a cotton thread during the cultivation under static condition in an incubator at 30 °C for a period of 10 days; then 50 mL of citric acid buffer (0.1 M, pH 4.0) was added to extract extracellular enzyme for laccase determination. The residues obtained after cultivation were separated via vacuum filtration and oven-dried at 40 °C as the fungal-treated OTS for sorption study.

### Biomass characterization and enzyme activity assay

The fungal treated OTS obtained from above procedure was weighed at room temperature to calculate percent biomass loss and also subjected to chemical and physical properties analysis such as composition, surface area, and pore size (JW-BK100A, JWGB Co., Ltd., China). Laccase activity in the crude extract was determined at room temperature by recording the increase in absorption value at 470 nm using 2,6-Dimethoxyphenol (DMP, Sigma) as substrate^23^. The assay system (3 mL) contained 0.1 mL of the crude enzyme extract, 0.5 mL of 10 mM DMP, and 2.4 mL sodium citrate-phosphate buffer (0.1 M, pH 3.5). One unit of laccase activity represents the amount of enzyme transforming 1 μmol DMP in 1 min. Polyacrylamide gel electrophoresis (PAGE) was carried out with 5% stacking/12% resolving gels. After electrophoresis, gels were stained with DMP solution described above for 20 min to visualize laccase isozymes.

### SEM observation and FTIR spectra analysis

The surface morphology of the adsorbent OTS was recorded with scanning electron microscopy (SEM). The fourier transform infrared (FTIR) spectra of OTS were obtained in the range of 4000–400 cm^−1^ using a fourier transform infrared spectrometer (VECTOR 22, Bruker, Germany).

### Adsorption measurement and isotherm

Three types of OTS with or without fungal pretreatment were used for adsorption of dye MB. To study the effects of some parameters, namely pH, dye concentration, contact time, and temperature, batch experiments were carried out on a rotary shaker (BJPX-Kansas) at 150 rpm and 25 °C using 100 mL flasks containing 50 mL of the respective dye solutions and a known amount of the adsorbent. The flasks were sealed with parafilm to avoid changes in the solution volume during the experiments. After certain time intervals, 4 mL solution was sampled from the flasks and then centrifuged (BECKMAN J2-MC, USA) at 5000 rpm for 10 min. Dye concentrations in the supernatant solutions were estimated by measuring the absorbance at the wavelength of 663 nm using a spectrophotometer (VIS-7220, Beijing, China) as stated above. All the experiments were carried out in triplicate and the standard deviations were below 3%. The amount of dye MB sorbed by OTS was calculated using the following equation:


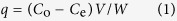


where *q* (mg/g) is the amount of dye sorbed by biosorbent; *C*_o_ and *C*_e_ (mg/L) are the initial and equilibrium concentration of the dye, respectively; *V* (L) is the initial volume of dye solution; and *W* (g) is the weight of the biosorbent.

The Langmuir and Freundlich equations were employed to study the sorption isotherms of dye MB^2^. The linearized form of the Langmuir equation is as follows:


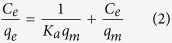


where *K*_α_ (L/mg) is the Langmuir adsorption constant and *q*_m_ (mg/g) is the maximum amount of adsorption corresponding to complete monolayer coverage on the surface, *q*_e_ (mg/g) is the amount of dye adsorbed by sorbent at equilibrium, and *C*_e_ (mg/L) is the equilibrium concentration of dye solution. The separation factor *R*_*L*_ was calculated using the following equation where *C*_*o*_ is the initial dye concentration:


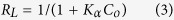


The linearized Freundlich equation is as follows:


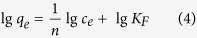


where *K*_*F*_ is an indicator of adsorption capacity (mg/g) and 1*/n* is the adsorption intensity.

The change in free energy (Δ*G*°) was evaluated using the following equation to study the thermodynamic nature^7^:


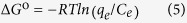


where *R* is the gas constant (8.3143 J/mol K), and *T* is the absolute temperature.

### Adsorption kinetics

The above kinetic data under various contact times were treated with the following Lagergren’s pseudo first-order (6) and Ho’s pseudo second-order rate (7) equation:


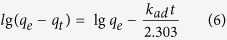



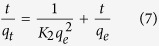


where *q*_*e*_ and *q*_*t*_ (mg/g) refer to the amount of dye sorbed at equilibrium and time *t* (min), respectively, and *K*_*ad*_ (/min) and *K*_2_ (g/mg·min) are the rate constants.

## Results and Discussion

### Fungal treatment of OTS

**Figure 2:**
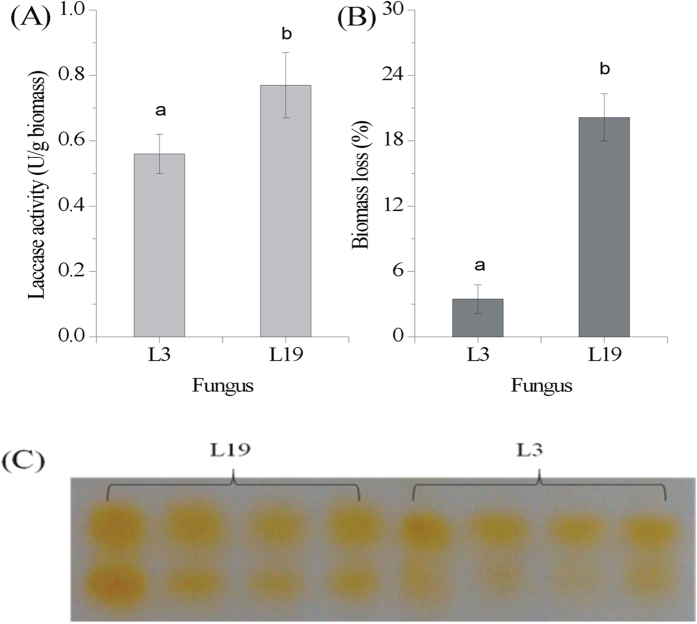
Laccase production (**A**), OTS biomass loss (**B**), and Native-PAGE of laccase activity (**C**) by two fungi.

Fungal solid state cultivation was performed on OTS, where laccase activity and OTS biomass loss were investigated. As shown in Fig. 2A, *Trametes* L19 was superior to *Pycnoporus* L3 in extracellular laccase production in terms of using OTS as medium, yielding 0.67 U/g and 0.56 U/g enzyme, respectively, showing the differed capabilities of enzyme production among various fungi on the same culture medium^12^. The treatment with fungus L19, however, resulted in a higher loss of OTS biomass than that by L3. Specifically, 3% of OTS was consumed by L3 and 20% by L19 after fungal cultivation (Fig. 2B). With active dying of laccase with DMP as substrate on Native-PAGE, two isozymes were visualized from both the fungi (Fig. 2C). From the electrophoretogram, laccase activity by fungus L19 was observed higher than that by L3. Under submerged cultivation, these fungi normally secrete more than two isozymes^20^,^21^, exhibiting a stronger capability in laccase production than that for cultivation on sole OTS. The generated laccase-containing extract may serve as a beneficial co-product with potential applications elsewhere, such as alleviation of turfgrass soil water repellency^15^ and dye decolorization^24^. Biosorbent modifications are often thought to increase the total cost^2^; however, fungal treatment can produce value-added enzymes as a compensation to some degree.

Chemical composition and physical properties of the raw and fungal treated OTS were characterized (Table 1). Raw OTS contained approximately 2.69% crude protein, 18.15% crude cellulose, 3.60% crude fat, and 3.44% crude ash, whereas both L3 and L19 treated OTS had a higher content in three nutrition, but lower in crude ash. Apparently, OTS has less cellulose component relative to other lignocellulosic materials reported^25^. Fungal metabolism can lead to increased nutritive value in biomass and meantime also simultaneously result in more solubility of ash trapped and/or attached on biomass surface^11^. Regarding the physical properties of OTS, fungal treatment also showed significant enhancement in specific surface area (SSA) and average pore size but weak effect on total pore volume (Table 1). The most dramatic enhancement was observed in SSA, reaching almost 50% after fungal treatment (from 0.535 m^2^/g to 0.807 m^2^/g).

### Adsorption of MB and isotherm

**Figure 3:**
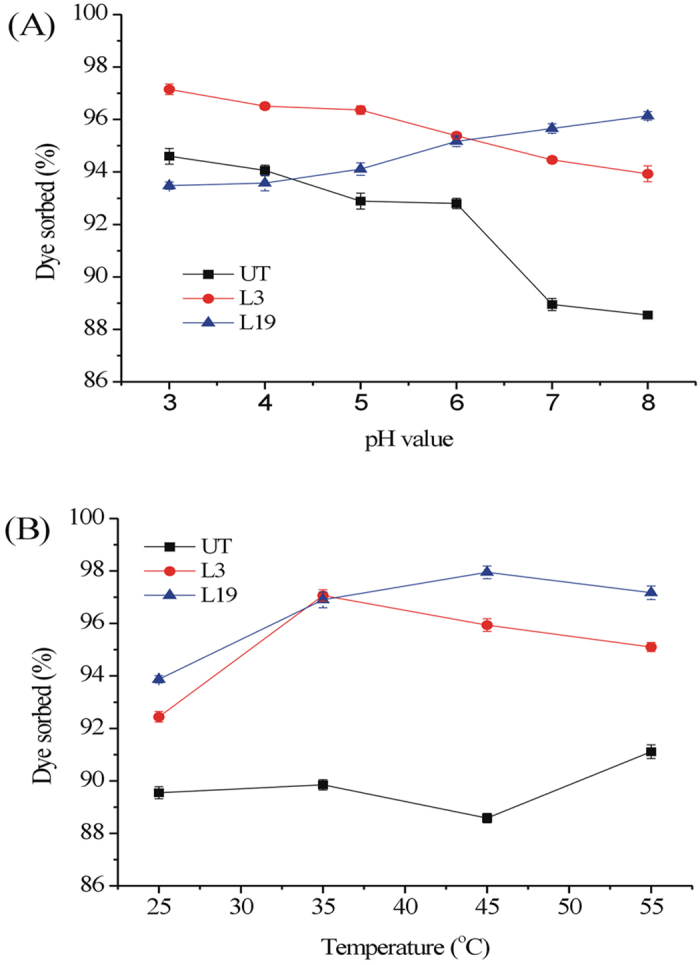
(**A**) Effect of pH on adsorption of MB by OTS (initial dye concentration: 50 mg/L; sorbent dose: 2 g/L; particle size: 10 mesh; contact time: 24 h; temperature: 25 °C). (**B**) Effect of temperature on adsorption of MB by OTS (initial dye concentration: 50 mg/L; sorbent dose: 2 g/L; particle size: 10 mesh; contact time: 24 h; pH: 6.5).

**Figure 4:**
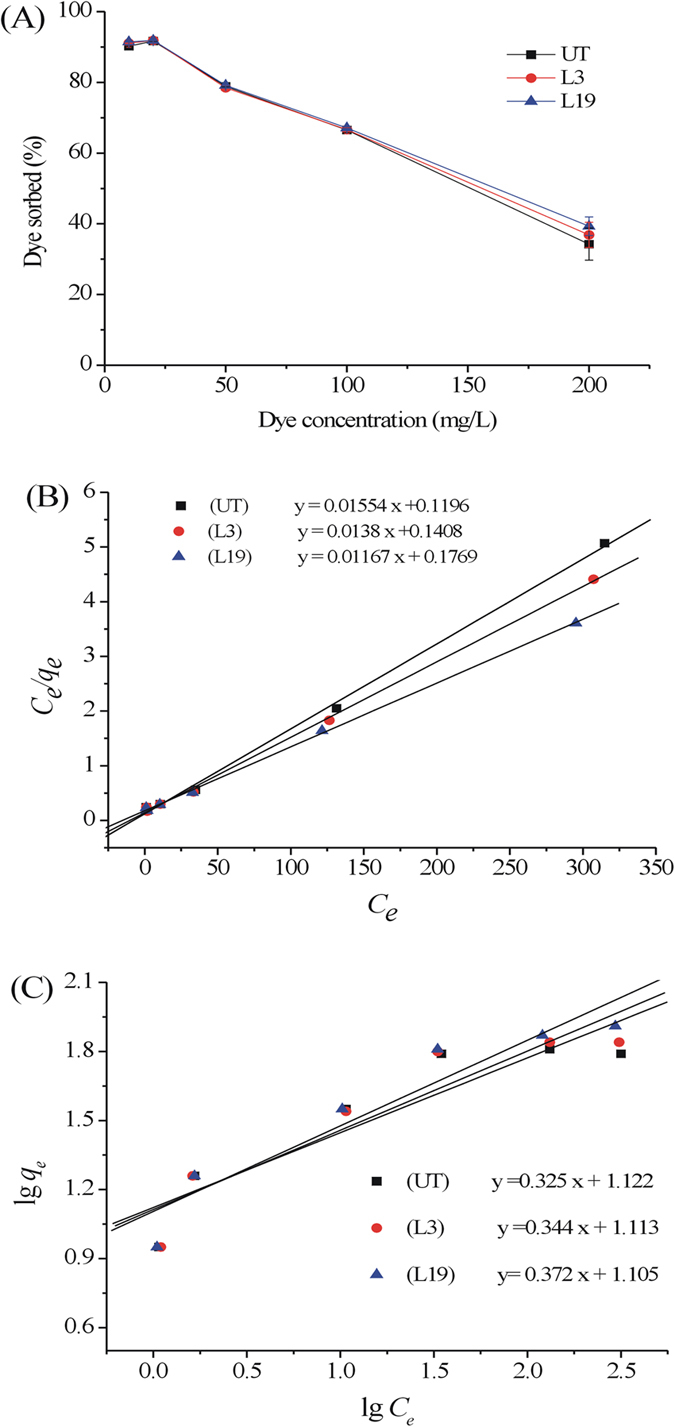
(**A**) Effect of dye concentration on adsorption of MB by OTS (sorbent dose: 2 g/L; particle size: 10 mesh; contact time: 24 h; pH: 6.5; temperature: 25 °C). Langmuir (**B**) and Freundlich (**C**) isotherms for MB adsorption on untreated and fungal treated OTS.

**Figure 5:**
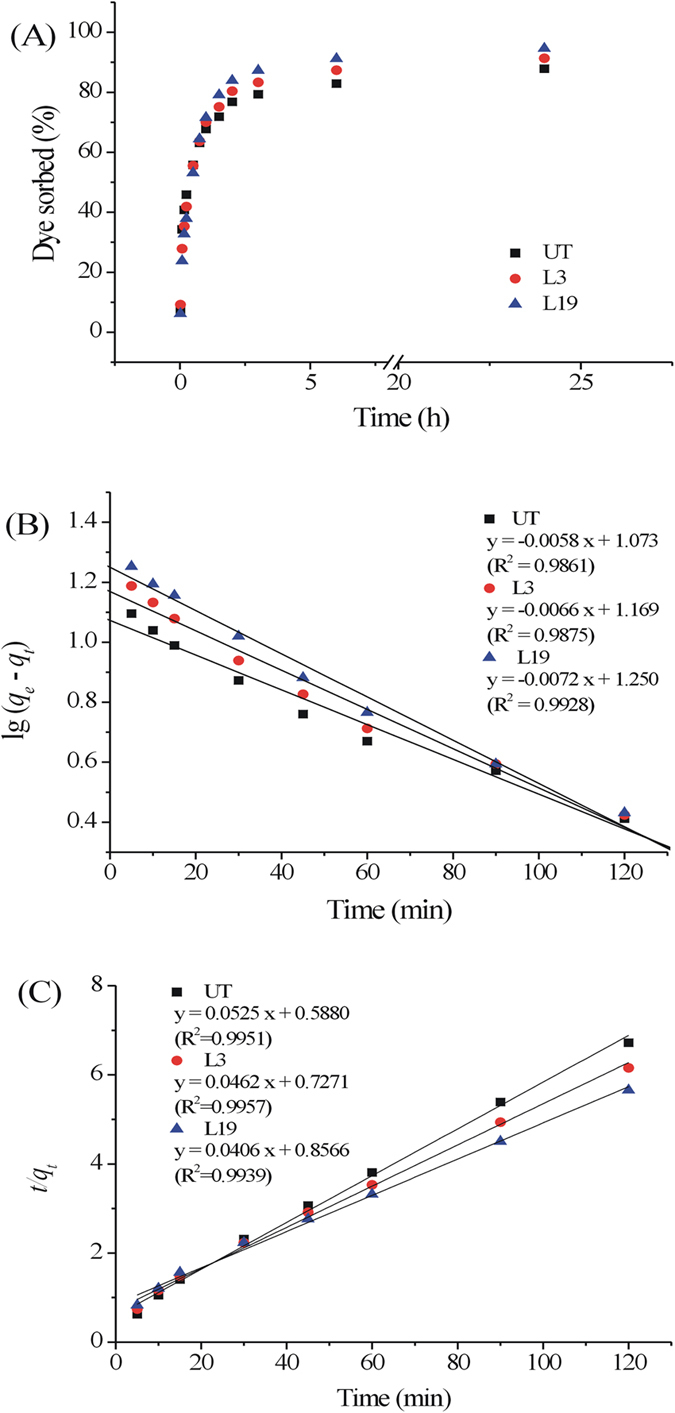
(**A**) Adsorption kinetics for MB by OTS (initial dye concentration: 50 mg/L; sorbent dose: 2 g/L; particle size: 10 mesh; pH: 6.5; temperature: 25 °C). Pseudo first order model (**B**) and Pseudo second order model (**C**) for adsorption of MB by OTS.

Both the raw OTS (UT) and the fungal treated ones were employed as adsorbents to remove dye MB from aqueous solution (Figs 3, 4 and 5). As solution pH increased, the biosorptive removal of MB was enhanced by L19 treated OTS, but reduced by raw OTS and L3 treated OTS (Fig. 3A). The best dye removal occurred at pH 3 for UT and L3, while the maximum dye sorption was observed at pH 8 for L19. This suggests that fungal treatment might alter the electric charge distribution on the surface of OTS. In the range 25–55 °C, raw OTS did not show strong difference in MB sorption, whereas fungal treated OTS showed an increase and then decrease in biosorption capacity with the best temperature ranging from 35 to 45 °C (Fig. 3B). Obviously, fungal treatment altered dye sorption by OTS under different pH and temperature conditions. Among these influencing factors, pH and temperature are the most important regulators of the sorption process.

As shown in Fig. 4A, when the dye concentration was increased from 10 to 200 mg/L, the percentage of dye sorbed initially increased and then decreased from approximately 91% to <40%. The highest dye removal rate was found to be around 93% at MB concentration of 20 mg/L with *q*_*e*_ of 23.5 mg/g achieved. When the dye concentration reached 100 mg/L, only 65% of MB was removed from aqueous solution with *q*_*e*_ of only 10 mg/g, partially due to the saturation of adsorption sites. Hassan *et al*. reported an increased MB uptake when more contact time and higher pH value and dye concentration were applied^26^, slightly different from our results.

The sorption data were fitted with two adsorption isotherms, Langmuir and Freundlich models (Fig. 4B,C) and the related sorption parameters are summarized in Table 2. Langmuir model for three OTS materials described equilibrium better (*R*^2^ > 0.99) than Freundlich model (*R*^2^ < 0.95) over the range of MB concentrations studied, indicating that MB sorption was more inclined to occur as mono layer than multiple layers on OTS. The monolayer adsorptive capacity (*q*_m_) by raw OTS was 64.35 mg/g and increased to 72.46 mg/g and 85.69 mg/g for L3 and L19 treated ones, respectively. Although better adsorption was been achieved for OTS after fungal cultivation, the treatment cost and long duration should be taken into account for practical applications. Langmuir constant (*K*_*α*_) was calculated to be 0.130, 0.098, and 0.066 L/mg for raw OTS, L3, and L19 treated OTS (Table 2), indicating sorption affinity of MB to OTS was decreased with fungal treatment^27^. Separation factor *R*_*mL*_ was 0.191, 0.231, and 0.296 for raw, L3, and L19 treated OTS, indicating that adsorption was favorable (0 < *R*_*L*_ < 1)^28^. In the Freundlich model, values of 1/n for three types of OTS were all <1, suggesting the biosorption was very feasible as well^22^.

The change in free energy (Δ*G*°), one criterion of spontaneity, was evaluated at different temperatures and the results are given in Table 3. The calculated Δ*G*° (−3500 J/mol to −8450 J/mol) was far below zero for all OTS sorbents to adsorb dye MB at four given temperatures, evidencing the spontaneous nature of MB adsorption on OTS^2^. Similarly, Hassan *et al*. reported Δ*G*° value of around −4000 J/mol at 25 °C for dye MB sorption on *Haloxylon recurvum* plant stems (HRS)^26^.

### Adsorption Kinetics

The biosorption kinetics of dye MB on OTS are illustrated in Fig. 5. The removal rates of MB were very rapid during the initial stage (within 2 h) of the biosorption process by three different biomasses (Fig. 5A). At 3 h, dye removal rates by UT, L3, and L19 treated OTS were 80%, 84%, and 89%, respectively. After a very rapid biosorption, dye uptake increased slowly with elongation of contact time and reached equilibrium values at approximately 3 h for all three biomasses. The final dye removal rates after 24 h by UT, L3, and L19 treated OTS were 88%, 92%, and 96%, respectively. Fungal pretreatment did not shorten or prolong the time required for adsorption equilibrium but increased dye removal rate for a certain contact time. The rapid biosorption during the initial stages demonstrated that dye binding on OTS was physical sorption.

The above kinetics data were treated with the Lagergren’s pseudo first-order equation and Ho’s second-order equation (Fig. 5B,C). As can be seen from correlation coefficient (*R*^2^ > 0.99), pseudo second-order equation well described the kinetics of adsorption process. The *k*_*2*_ values calculated from the slope of the linear plots of MB adsorption were 0.005, 0.003, and 0.002 g/mg per min for UT, L3, and L19 treated OTS, respectively (Table 4). The predicted *q*_*e*_ for three OTSs were 19.05, 21.65, and 24.63 mg/g, very close to the experimental data 22.25, 23.00, and 23.75 mg/g, while the data obtained with the pseudo first-order equation differed greatly with the experimental data (Table 4).

MB is a widely used toxic cationic basic dye, and its removal from aqueous solution by adsorbents has been previously studied^1^. Rice husk ash (RHA) removed over 99.9% MB from aqueous phase and the adsorption followed the Temkin isotherm and pseudo second-order kinetics^29^. HRS adsorbed MB with *q*_m_ of 22.93 mg/g^26^, far lower than that observed in this study by OTS. Various modifications of biomass have been conducted for improving dye adsorption has been attempted in published literatures. Hu *et al*. modified pummelo peel with NaOH, achieving 390.6 mg/g (*q*_*m*_) relative to the untreated peel (*q*_*m*_ = 170.6 mg/g)^8^. Bedin *et al*. synthesized activated carbon (AC) from sucrose using hydrothermal and subsequent KOH treatments, achieving *q*_*m*_ of 704 mg/g for dye MB with surface area of AC enlarged up to 1534 m^2^/g^30^.

### SEM observation of OTS

**Figure 6:**
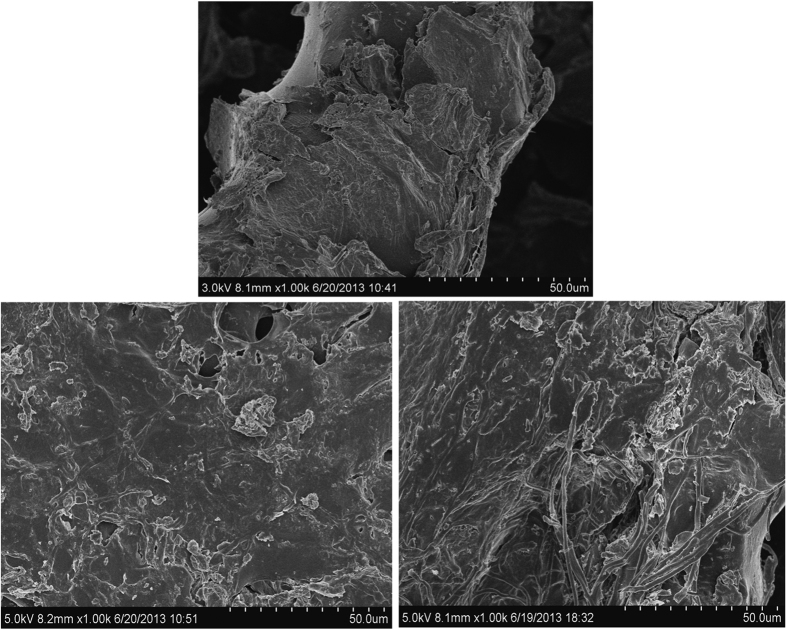
The SEM observation of untreated (upper) and fungal treated OTS (lower left: fungus L3; lower right: fungus L19).

Figure 6 presents the SEM micrographs of untreated and fungal treated samples. Raw materials showed a rough surface morphology and various pores in different sizes; these further intensified after fungal treatment, forming more pores and pits on the surface of OTS, very similar to that observed in fungal pretreated switchgrass^10^. This virtually increased the surface area of biosorbent and thus enlarged the contact area with dye MB (Table 1). The pores coupled with rough surface are believed to be important for dye sorption^22^. Various fungal biomasses have been found to be effective adsorbents for dye removal^31^,^32^,^33^. However, because fungal treated OTS had been extracted with phosphorus buffer for extracellular enzymes, mycelia leftover was therefore simultaneously removed from the treated OTS (Fig. 6) and the enhanced adsorptive capacity of OTS should be mainly attributed to fungal modification.

### FTIR spectra of OTS

**Figure 7:**
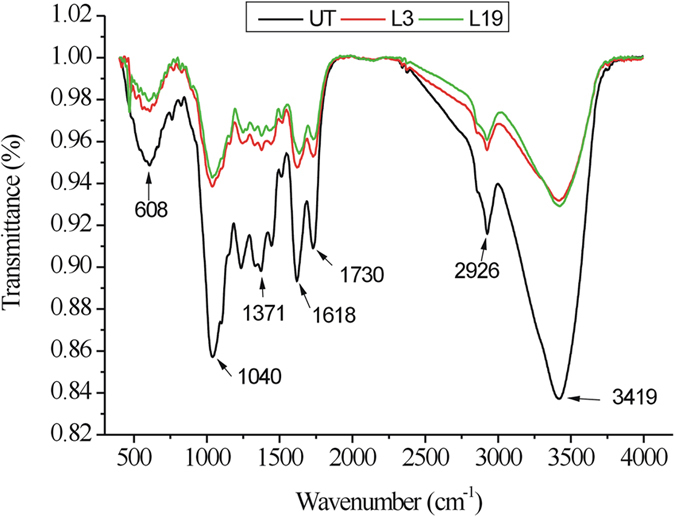
The FTIR spectra of untreated (UT) and fungal treated OTS.

To better understand the underlying adsorption mechanism between MB and the functional groups of OTS, FTIR spectra of OTS with or without fungal treatment were obtained and the results are depicted in Fig. 7. The changes in FTIR spectra between raw and fungal treated OTS evidenced the successful modification. Specifically, FTIR characteristic of OTS showed several typical absorbance peaks ranging from wavelength 4000 to 400 cm^−1^, corresponding to 3419, 2926, 1040–1730, and 608 cm^−1^, respectively. These absorption peaks indicate the polyfunctional groups on the surface of OTS^26^. Fungal modification apparently decreased the absorbance intensity at these wavelengths, probably partially responsible for enhanced dye adsorption. Similarly, the intensity of peaks of sawdust was decreased after formaldehyde treatment^34^. However, no remarkable discrepancies were observed between L3 and L19 treated OTS. By comparison, it can be found that the changes in FTIR spectra in this study were more significant than those in chemically modified lignocellulosic materials^8^,^35^.

## Conclusion

This study suggests that the agro-waste oiltea shell (OTS) can be a novel inexpensive biosorbent for dye removal from aqueous solution, and fungal treatment is an effective method to enhance sorptive performance and simultaneously produce extracellular enzymes with potential applications. The underlying mechanism governing the sorptive enhancement is associated with the changes in chemical composition, physical properties, and also the functional groups in the fungal treated OTS. To maximize sorptive enhancement, optimization of cultivation should be conducted among different lignocellulosic biomasses using different fungi in the future.

## Additional Information

**How to cite this article**: Liu, J. *et al*. Adsorption of methylene blue on an agro-waste oiltea shell with and without fungal treatment. *Sci. Rep.* **6**, 38450; doi: 10.1038/srep38450 (2016).

**Publisher's note:** Springer Nature remains neutral with regard to jurisdictional claims in published maps and institutional affiliations.

## Figures and Tables

**Table 1:** Characteristics of untreated and fungal treated OTS.

OTS	Chemical composition				Physical properties		
	CP (%)	CC (%)	CF (%)	CA (%)	SSA (m^2^/g)	TPV (cm^3^/g)	APS (nm)
Untreated	2.69^a^	18.15^a^	3.60^a^	3.44^b^	0.535	0.002	15.947
L3 treated	3.26^b^	23.10^b^	4.46^b^	2.25^a^	0.807	0.002	16.318
L19 treated	3.60^c^	24.60^c^	4.73^c^	2.32^a^	0.778	0.003	16.502

CP: crude protein CC: crude cellulose CF: crude fat CA: crude ash SSA: specific surface area TPV: total pore volume APS: average pore size.

**Table 2:** The Langmuir and Freundlich model parameters for biosorption of MB on OTS.

OTS	Langmuir				Freundlich		
	*q*_*m*_ (mg/g)	*Kα* (L/mg)	R^2^	*R*_*mL*_	*K*_*F*_	*1/n*	R^2^
Untreated	64.35	0.130	0.9990	0.191	13.24	0.325	0.9206
L3 treated	72.46	0.098	0.9994	0.231	12.97	0.344	0.9342
L19 treated	85.69	0.066	0.9996	0.296	12.73	0.372	0.9524

(T = 25 °C, *R*_*mL*_ is the average of equilibrium parameters measured at the initial concentrations).

**Table 3:** Free energy change (Δ*G*°) for the biosorption of MB on OTS at different temperatures.

OTS	Free energy change (J/mol)			
	25 °C	35 °C	45 °C	55 °C
Untreated	−3591.9	−3853.3	−3563.1	−4419.9
L3 treated	−4509.1	−7217.3	−6573.3	−6141.5
L19 treated	−5101.6	−7130.2	−8459.3	−7592.9

**Table 4:** Parameters for Lagergren pseudo first order and Ho’s pseudo second order kinetic models for the adsorption of MB on OTS.

OTS	Pseudo first order kinetic model		Pseudo second order kinetic model		Experimental
	*K*_*ad*_ (/min)	*q*_e (cal)_ (mg/g)	*K*_*2*_ (g/mg·min)	*q*_e (cal)_ (mg/g)	*q*_e (exp)_ (mg/g)
Untreated	0.013	11.83	0.005	19.05	22.25
L3 treated	0.015	14.76	0.003	21.65	23.00
L19 treated	0.016	17.78	0.002	24.63	23.75

(T = 25 °C, C_o_ = 50 mg/L).
