# Erratum: Adsorption of methylene blue on an agro-waste oiltea shell with and without fungal treatment

**DOI:** 10.1038/srep44160

**Published:** 2017-03-16

**Authors:** Jiayang Liu, Enzhong Li, Xiaojuan You, Changwei Hu, Qingguo Huang

Scientific Reports 6: Article number: 3845010.1038/srep38450; published online: 12 05 2016; updated: 03 16 2017

In this Article, Enzhong Li is incorrectly listed as being affiliated with ‘Shandong Provincial Key Laboratory of Water and Soil Conservation and Environmental Protection, Linyi University, Linyi, 276000, China’ The correct affiliation is listed below:

Fermentation Technology Division, School of Bioengineering, Huanghuai University, Zhumadian 463000, China.

